# Development of a novel voltammetric method for revefenacin determination using modified electrodes in pharmaceutical and biological matrices

**DOI:** 10.1186/s13065-026-01757-6

**Published:** 2026-03-27

**Authors:** Sherin F. Hammad, Hala M. Habib, Hassan A. Hendawy

**Affiliations:** 1 Pharmaceutical Analytical Chemistry Department, Faculty of Pharmacy, Tanta University, Elgeish street, Tanta, Egypt; 2 Egyptian Drug Authority (EDA), Giza, Egypt

**Keywords:** Revefenacin, Electrochemical sensors, Surfactants, Nanomaterials, Biological matrices

## Abstract

Revefenacin (REV) is an inhalation solution used for management of Chronic Obstructive Pulmonary Disease (COPD) in the USA, this is single medication that is an approved bronchodilator delivered by nebulization with a once-daily frequency. Even though the crucial necessity of quality control in pharmaceutical manufacturing, no officially recognized methods exist for REV determination in biological samples. A sensitive, economical, simple and efficient electrochemical procedure using carbon paste modified with nanostructured zinc oxide electrode (ZnO–NPs/CPE) was designed for remarkably trace analysis of REV. It was demonstrated that the (ZnO–NPs/CPE) electrode in the Britton–Robinson buffer adjusted to pH 4.0 with the anionic surfactant sodium dodecyl sulfate (SDS), the electrochemical oxidation of REV was significantly enhanced. Under optimal conditions, the REV electrochemical process was irreversible and adsorption-mediated. Scanning Electron Microscopy (SEM) and Energy-Dispersive X-ray Spectroscopy (EDX) were utilized to characterize the morphology of the modified electrode. Under optimal experimental conditions, the analytical results showed direct relationship responses in the range of 0.19–2.0 µg mL^−1^ and a low limit of detection (LOD) of 0.04 µg mL^−1^ and limit of quantification (LOQ) of 0.13 µg mL^−1^. Our investigation reveals the outstanding applicability of the suggested sensor in quality control laboratories to achieve the fast determination for REV across various sample types (pure form, dosage form, biological samples).

## Introduction

**Fig. 1:**
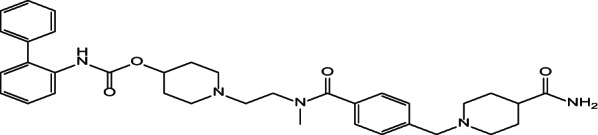
Structure of REV

REV drug is a white powder, slightly soluble in water but freely soluble in acetonitrile and in methanol. The IUPAC name for REV is [1-[2-[[4-[(4-carbamoylpiperidin-1-yl) methyl] benzoyl]-methylamino] ethyl] piperidin-4-yl] N-(2-phenylphenyl) carbamate [1], as illustrated in Fig. 1. It is known that REV offers prolonged bronchodilation effect [2]. REV inhalation solution YUPELRI^®^ [3] is a once-daily, lung-selective administered using a standard jet nebulizer [4]. There are limited numbers of reports on quantitative analytical techniques for REV determination in biological fluids and pharmaceutical formulations such as chromatographic methods (RP-HPLC) [5] and UHPLC [6]. There is a critical need for a simple, rapid and sensitive alternative techniques. Voltammetric techniques are highly favored for this purpose due to their simplicity, portability, low cost, minimal maintenance and reduced requirement for organic solvents. Therefore, the development of a simple, novel and first-ever investigation of the electrochemical behavior and a fully validated electroanalytical method for REV is useful alternative to sophisticated and time consuming HPLC methods. Furthermore, theoretical analysis of the REV chemical structure suggests that it is an electroactive molecule capable of straightforward electrochemical oxidation.carbon paste electrodes (CPEs) are popular materials [7]due to their consistent performance, ease of modification with various substances, ability to produce reliable measurements and the availability of affordable components [8]. Nanoparticles [9], possessing unique physical, chemical and electrical characteristics and exhibiting nanoscale dimensions (typically 1–100 nm) are employed in a wide variety of analytical procedures including voltammetric techniques. Zinc oxide nano particles [10] (ZnO-NPs) possess great value as of their utilization in biosensors, cosmetics and also drug delivery systems. ZnO-NPs show a pronounced electro-catalytic effect coupled with high adsorption affinity [11].The proposed method is depended on using the ZnO-NPs/CPE sensor [12] which optimizes the sensitivity of the method with relatively low potential as they exhibit a large surface area and increases electrocatalytic activity. Surfactants are crucial in electrochemical processes due to their ability to reduce surface tension and modify electrode’s surface features. This modification enhances the interaction between the electrode and electroactive species. Our study employed anionic sodium dodecyl sulfate (SDS), cationic cetyltrimethylammonium bromide (CTAB) surfactants. This study pioneered a novel first-ever electrochemical technique for measuring REV in pure form, commercial products and biological samples using a ZnO-NPs/ CPE. The modified electrode was characterized using SEM and EDX. Differential pulse voltammetry (DPV) served as the technique for exploring modified electrode’s interaction with the electroactive species and their resulting electrochemical behavior.

## Materials and methods

### Instrumentation

The measurements were conducted using a Metrohm (797 VA Electro-analyzer) operating with Computrace software (version 1.3.1) for voltammetric investigations. The electrode setup was composed of working electrode, Ag/AgCl (3.0 M potassium chloride) was used as a RE (reference electrode) and a platinum wire functioned as auxiliary electrode. JENWAY pH-meter was appropriated for pH determinations. AMETEK (EDAX) model: OCTANE PRO, electronic microscope MODEL: QUANTA FEG250. A Sartorius balance and micropipette were used throughout the present experimental work. The entire set of experiments took place at a controlled temperature of (25 ± 0.1 °C).

### Reagent and chemicals

Deionized water was employed throughout the investigation. The Egyptian drug authorities provided the REV reference standard, orthophosphoric acid (1.05573), glacial acetic acid (Ac20181150), boric acid (2341), CTAB, sodium chloride isotonic solution 0.9%, citric acid, sodium citrate, SDS and graphite powder. Merck supplied the paraffin oil (76235), sodium hydroxide (22145) and zinc oxide nanoparticles (677450). Fisher Scientific supplied methanol (Steinheim, UK). The urine sample was donated from healthy volunteers.

### Preparation of electrolyte solution

Britton–Robinson (B-R) buffer 0.04 M was elaborated by using Boric acid, orthophosphoric acid and glacial acetic acid [13]. Subsequently, the B-R buffer was adjusted to the pH range from (2–12) with 0.2 M sodium hydroxide.

### Preparation of REV standard stock solution

In order to prepare 0.6 mg/mL (1 × 10^− 3^ M) REV reference standard stock solution, 15 mg of REV were dissolved in 2 mL methanol in a 25 mL measuring flask, then complete to the mark with distilled water, appropriate volume of this solution was transferred to 25- mL volumetric flask and then completed to the mark with distilled water in order to obtain (1 × 10^− 4^ M) reference standard stock solution. The obtained stock solutions were prepared immediately before testing.

### Preparation of stock solutions of the surfactants

The stock solutions of SDS and CTAB were prepared by accurately dissolve 14.4 mg and 18.2 mg respectively with 50.0 mL distilled water in a measuring flask to achieve the desired molar concentration (1 × 10^− 3^M) for each compound. The solutions were freshly prepared just before the tests.

### Preparation of isotonic solution pH 5

To Prepare 0.1 molar stock solution of citric acid precisely 2.101 g was dissolved in water in 100 mL volumetric flask and complete the volume with water. To prepare 0.1 M trisodium citrate solution precisely weight 2.941 g in 100 mL volumetric flask and complete the volume with water. Accurately combine 35 mL of the 0.1 M citric acid stock with 65 mL of the 0.1 M trisodium citrate stock to initiate buffer formation. Verify and precisely adjust the solution’s pH to 5.0 using incremental additions of either stock solutions to achieve the desired pH, after that appropriate volume of this buffer was added to 100 mL sodium chloride isotonic solution to achieve pH 5 [14].

### Preparation of pharmaceutical samples

A mimetic REV inhalation solution with a concentration of 58.33 µg mL^−1^ was formulated in the lab by dissolving 5.8 mg REV in 100 mL volumetric flask with isotonic, sterile aqueous solution of sodium chloride precisely adjusted to pH 5.0 by citrate buffer. Appropriate aliquots of this solution transferred to 10 mL volumetric flask and completed to the mark with isotonic solution pH 5 to achieve 0.06 mg mL^−1^ (1 × 10^− 4^ M) pharmaceutical stock solution. Different volumes of this solution added to the voltammetric cell and follow the same procedures as under method of construction of calibration curve. differential pulse voltammograms were obtained. The derived regression equation was employed to estimate the resulting recovery.

### Preparation of biological sample

Urine specimens were provided by healthy volunteers, and 5 mL of the urine specimen combined with 0.3 mL of 5% zinc sulfate solution and 0.5 mL of ethanol. Afterward, the pH of the mixture was adjusted to eleven by using 1.0 molar sodium hydroxide solution creating an alkaline media to form a zinc hydroxide precipitate, it has a relatively large surface area and acts as an effective scavenger for proteins and other high-molecular-weight compounds present in urine [15]. The mixture was subsequently separated by centrifugation for fifteen minutes at 13 thousand revolutions per minute.

Then, filter the supernatant through 0.45 mm filter paper after that one mL of the supernatant spiked with different concentrations of REV stock solution (1 × 10^−4^ M) was added to voltammetric cell containing 14 mL of B-R buffer at pH 4.0 [16]. 

### Working electrodes Preparation

The CPE was fabricated by blending 0.5 g of graphite powder with a small amount of paraffin oil 0.3–0.5 mL to form a homogeneous paste [17].

ZNO–NP/CPE electrode [18]: It was made by homogenizing 0.5 g of graphite powder and an appropriate weight of (5% ZNO–NPs) powder then mixing well with 0.1 g paraffin oil for three min to obtain homogenous paste.

These pastes were then compressed into an insulin syringe with inner diameter of 3.0 mm, which held a copper wire to facilitate electrical current. A final smoothing step was conducted by pressing the electrode face against weighing paper.

### Establishing the calibration graph

The voltammetric behavior of REV at the ZNO-NPs/CPE were evaluated using 0.04 M B-R buffer adjusted at pH 4.0 in the presence of anionic surfactant SDS. Appropriate quantities of REV standard stock solutions 1 × 10^− 4^ M were added to a voltammetric cell contain 15 mL buffer and 0.75 mL SDS to achieve a final concentration range of 0.19–2.0 µg mL^−1^ of REV and the ZNO-NPs/CPE electrode was immersed in it. voltammograms were measured at a sweep rate of 60 mVS^−1^ and within the potential range from + 0.5 to + 1.4 V versus an Ag/AgCl RE using a pulse amplitude of 50 mV and a pulse time of 0.04 s. A standard curve was generated by plotting the measured anodic peak current (Ip​ in µA) against the concentration of REV µg mL^−1^, which then allowed for the calculation of the linear regression equation.

## Results and discussion

### Investigating the REV electrochemical response across different electrodes

**Fig. 2:**
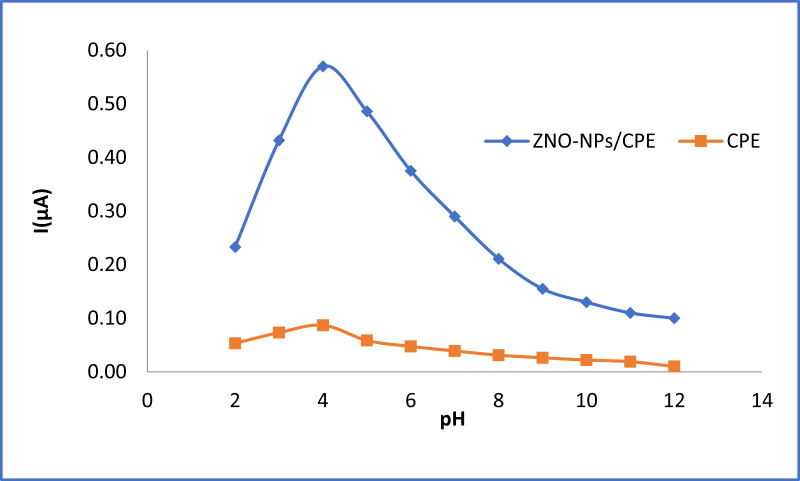
Voltammetric Comparison of REV 1 × 10 − 3 M behavior at pH rang (2–12) of CPE and ZNO-NPs/CPE working electrodes

The electrochemical characteristics of the REV molecule were established through the use of cyclic voltammetry and DPV with unmodified CPE and ZNO–NPs chemically combined with CPE. Differential voltammograms were obtained for 1 × 10^− 3^ M REV in buffer solution at pH range 2–12. The current strength of anodic peaks by ZNO–NPs/CPE was much higher than CPE (Fig. 2). Based on these findings, the ZNO-NPs/CPE was adopted as the optimal electrode throughout the study.

### Morphological differences among various electrodes

**Fig. 3:**
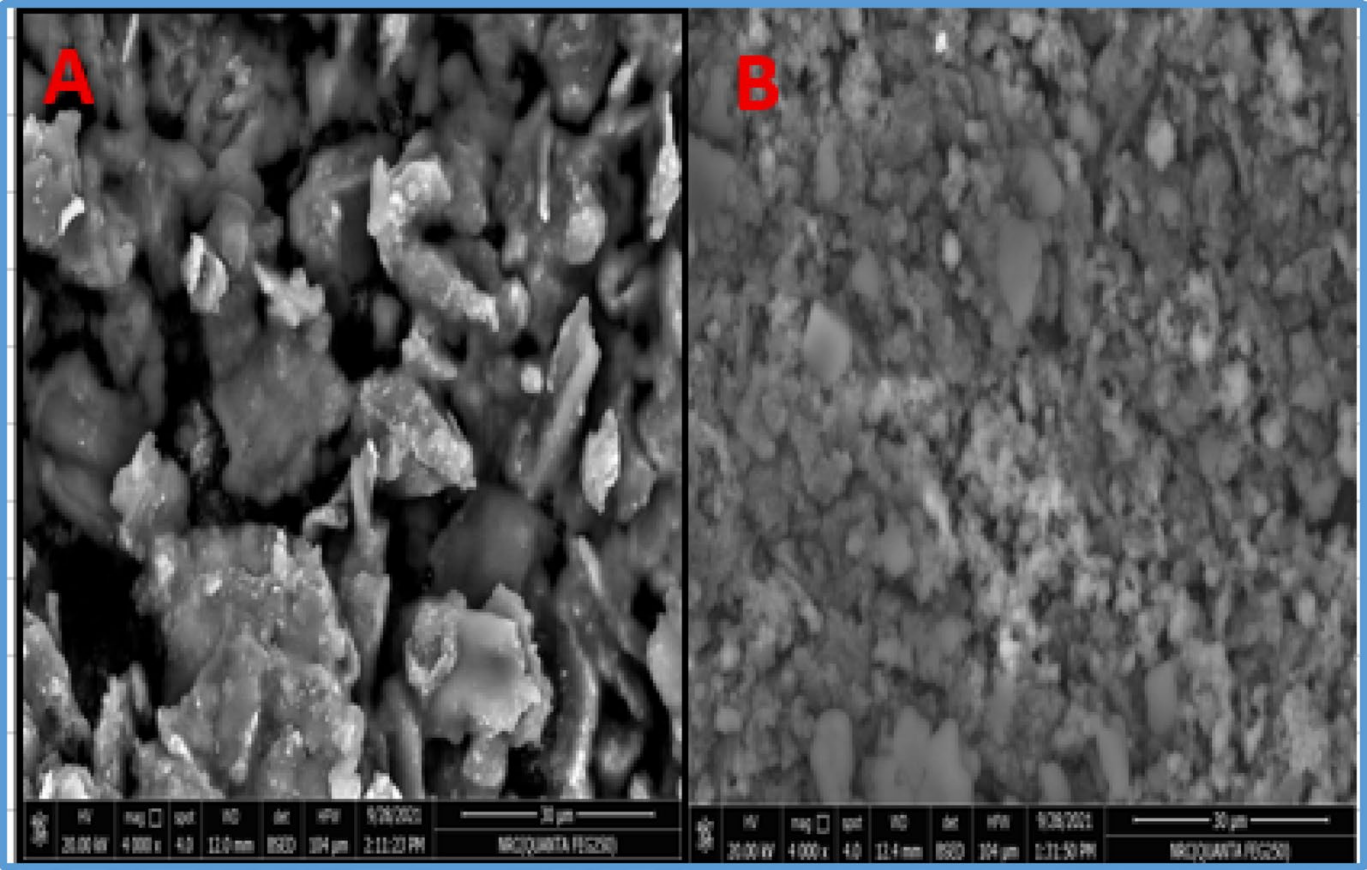
**A** SEM images of CPE, **B** ZnO–NPs/CPE, Magnification 4000 ×, Acceleration Voltage 20 KV, Working Distance (WD) 12.0 mm (**A**), 12.4 mm (**B**), Scale Bar 30 μm, Detector BRD (Backscattered Electron Detector)

**Fig. 4:**
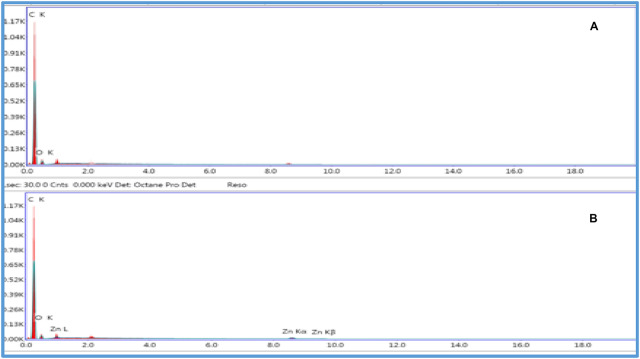
EDX images of CPE (**A**) and ZnO–NPs/CPE (**B**)

Structural analysis of unmodified CPE [19] and modified ZNO-NPs/CPE electrode [20] were examined using SEM images as illustrated in Fig. 3. The elemental analysis was performed using EDX as shown in Fig. 4. The effective blending of graphite particles with paraffin oil yields a highly compact paste, the SEM image of CPE surface typically appears as a collection of irregularly shaped, multi-layered graphite flakes or particles that are loosely packed and held together by the binder. The surface is generally rough and porous. The porosity comes from the spaces between the large, flaky graphite particles providing a relatively high surface area. The graphite particles might show some variation in size, resulting in a somewhat heterogeneous or separated surface where the graphite layers are distinct. Meanwhile, the modified electrode’s surface was markedly different, ZnO–NPs/CPE is a Nanoparticle clustering ranging in size ≤ 50 nanometers according to supplier specifications. The ZnO particles are typically well-dispersed and embedded or aggregated within the carbon matrix, confirming the modification process. The modification can result in a more uniform or denser layer covering the graphite, minimizing the spaces seen in the CPE. Elemental distribution within ZnO–NPs/CPE composite was investigated via EDX in the presence of zinc (Zn), oxygen (O) and carbon (C). SEM–EDX images qualitatively demonstrate the incorporation of zinc oxide nanoparticles within the carbon paste matrix. While surface roughness is not directly quantified, the addition of nanoparticles is consistent with literature reports in which comparable modifications lead to increased surface roughness and electrochemically active surface area [21].

### The electrochemically active surface areas of the electrodes

Cyclic voltammetry was employed to measure the electroactive surface area of every electrode, using a 1.0 mM K_4_Fe(CN)_6_ solution as a redox probe and measuring at different scan rates.

The surface area was calculated by applying the Randles–Sevcik equation as follow :

$${\mathrm{Ipa}} = {\text{ }}\left( {{\mathrm{2}}.{\text{69 }} \times {\mathrm{1}}0^{{\mathrm{5}}} } \right){\text{ n}}^{{{\mathrm{3}}/{\mathrm{2}}}} \times {\mathrm{A}} \times {\mathrm{C}}_{0} \times {\text{ D}}^{{{\mathrm{1}}/{\mathrm{2}}}} \times {\text{ }}\upsilon ^{{{\mathrm{1}}/{\mathrm{2}}}}$$

In this equation, Ipa represents the anodic peak current (µA), n is the number of electrons transferred, A is the surface area of the electrode (cm^2^), C_0_ is the concentration of K_4_Fe(CN)_6_ (mol/cm^3^), υ is the scan rate (V/s) and D is the diffusion coefficient which is 7.6 × 10^−6^ cm^2^/s for K_4_Fe(CN)_6_.

Given that *n* = 1 for the 1.0 mM K₄Fe(CN)₆ solution, the electrode surface area was determined from the slope of the plot of Ipa against υ^1/2^. The resulting calculated surface areas were 0.075 cm^2^ for CPE and 0.25 cm^2^ for the ZNO–NPs/CPE electrode. The improved electrochemical properties are primarily due to the introduction of ZnO–NPs, which expand the electroactive surface area and provide more pathways for electron transfer. This behavior is consistent with the well-established effect of nanomaterials on increasing the surface activity of carbon-based electrodes [22]. 

### Electrochemical characterization of REV as a function of pH using ZnO–NPs/CPE

**Fig. 5:**
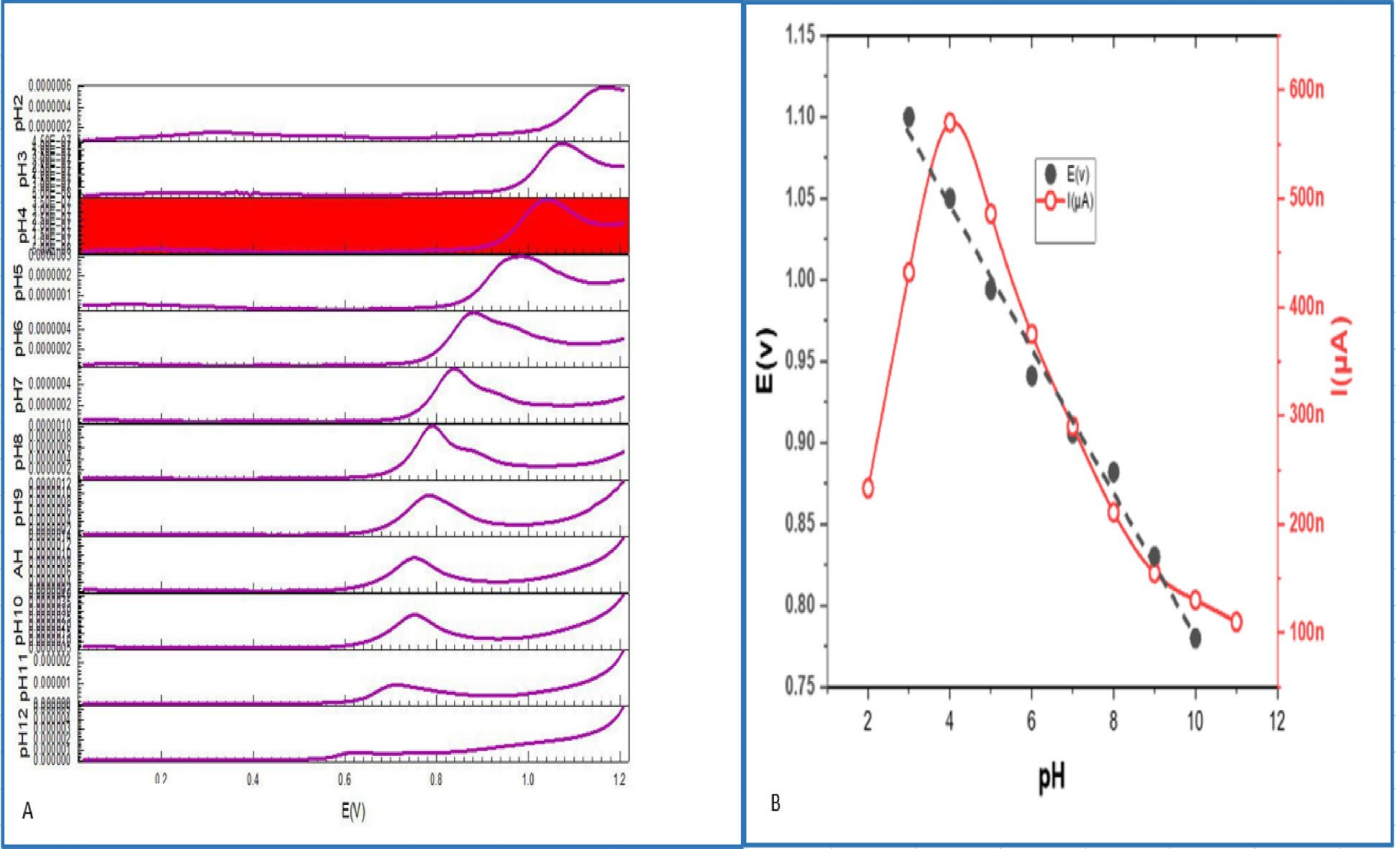
**A** The differential voltammetric response of REV at various pH values measured with ZnO–NPs/CPE at a scan rate of 100 mVs^− 1^. **B** comparison of the anodic peak currents and potential at various pH values using ZnO–NPs/CPE.

The electrode’s response exhibits a strong pH reliance of both buffering solution and immediate surrounding environment given both the oxidation potential and peak current are pH-dependent. A B-R buffer was employed covering the pH spectrum of 2 − 12 in order to determine how the pH of the solution impacts the electrocatalytic oxidation process of 1 × 10^− 3^ M REV. Plotting the peak current I(µA) as a function of pH revealed that the maximum peak current occurred at pH 4 (Fig. 5A). Additionally, using a plot of the anodic peak potential E(v) against pH values, it was seen that as the pH increased there was an inverse effect on the anodic peak potential (Fig. 5B). These results affirm the importance of protons in electrochemical processes. The regression line was calculated based on the subsequent formulation.

$$~{\mathrm{E}}\left( {\mathrm{V}} \right) = - 0.0{\text{358 pH}} + {\mathrm{1}}.{\mathrm{1666}}$$

with the coefficient of determination R^2^ was calculated to be 0.9967, indicating an excellent linear fit. The value 0.0358 V/pH or (35.8 mV/pH) is significant. It’s related to the theoretical Nernstian slope [23] for reactions involving proton transfer indicating unequal numbers of protons and electrons involved in the oxidation process [24]. 

### Investigating the electrochemical kinetics by changing the scan rate

To gather more data on how redox reactions occur at the modified electrode using cyclic voltammetry at pH 4. A scan rate analysis was conducted to determine how the scan rate influenced the current of the anodic peak [25]. The observed slope value of 0.9 and the theoretical value of 1.0 which is the expected value for a perfect response of surface species were closely identical. Consequently, this is validated by establishing the adsorptive characteristic of the process.

**Fig. 6:**
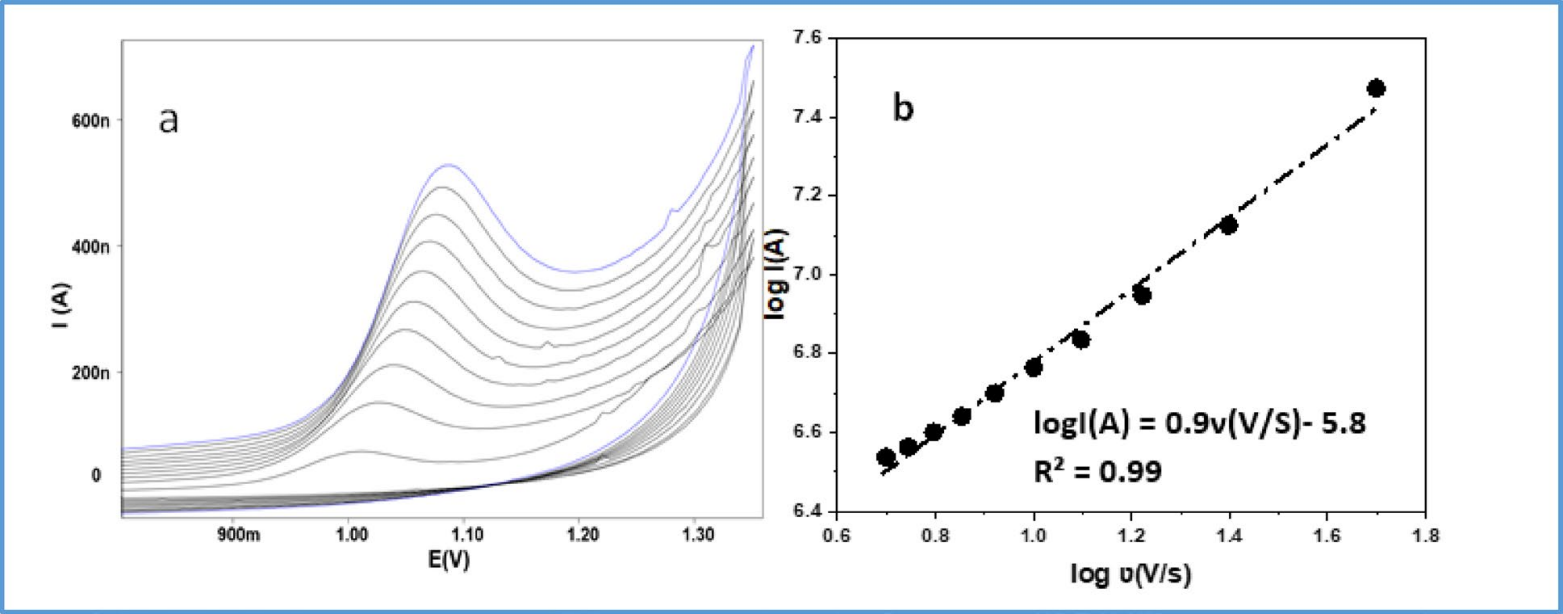
**a** Cyclic voltammogram of REV scan rate on ZnO–NPs/CPE in 0.04 M B-R buffer at pH 4.0 across the whole range from 20 to 400 mVs^−1^. **b** The relationship between the logarithm of scan rate and the logarithm of the anodic peak current of the REV

The anodic peak potentials shifted to the positive side with an increase in the currents, as shown in Fig. 6a, illustrating the irreversible character of the oxidation process. The following equations were obtained.

$${\mathrm{E}}\left( {\mathrm{V}} \right) = 0.0{\mathrm{7}}0{\text{3 log}}\nu {\text{ }}({\mathrm{vs}}^{{ - {\mathrm{1}}}} ) + {\mathrm{1}}.{\mathrm{1276}};{\text{ R}}^{{\mathrm{2}}} = {\text{ 99}}.{\mathrm{3}}$$

According to the Laviron theory [26] the slope of equation above can be used to directly determine the number of electrons involve in the reaction, the slope in these systems was 0.0703, In the case of a completely irreversible electrode process, alpha is often believed to be 0.5; hence, the number of electrons (n) transported during the oxidation of REV was determined to be 2. Also, the mechanism of the reaction of oxidation process at the electrode surface was confirmed by plotting log Ipa vs. log ν demonstrated a linear relationship (Fig. 6b).

### Voltammetric performance at different surface–active agents

To enhance the responsiveness and achieve ideal parameters, various surfactants: cationic surfactant CTAB and anionic surfactant SDS [27] were used and it was found that the CTAB eliminated the electrical current response as it inhibits oxidation reactions, resulting a substantial decrease in the oxidation rate which was linked to the creation of non-electrically conductive polymer structures across the electrode surface. Conversely the oxidation peaks in presence of SDS were markedly more pronounced than ZnO–NPs/CPE in lack of surfactant.

**Fig. 7:**
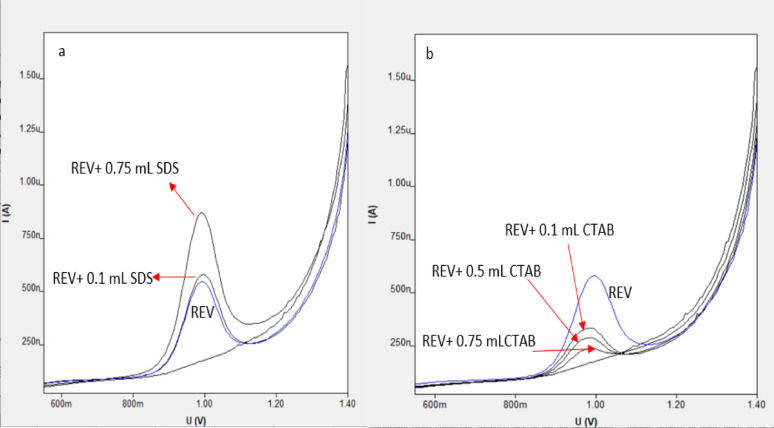
The effect of various surfactants, anionic surfactant SDS (**a**) and cationic surfactant CTAB (**b**) on the differential pulse voltammetric response of 1 × 10 ^− 4^ M of REV in B-R buffer at pH 4.0 on ZnO–NPs/CPE. *The first voltammograms with no signals is the blank

This because the REV is a cationic species due to the protonation of its nitrogen. The adsorbed SDS generates a negatively charged hydrophilic film on the electrode surface, with the anionic polar head groups facing the solution. This negatively charged layer creates an electrostatic attraction that effectively facilitates the rapid transport and accumulation of the positively charged REV species at the electrode surface, consequently accelerating the reaction rate The plot of the anodic peak current I(A) and potential E(V) at the electrode surface with modified doses of SDS shows an Incremental advancement in peak current with the greatest effect at 750 µL additional of SDS in buffer pH = 4 containing REV as illustrated in Fig. 7; Table 1. The use of surfactants in electrochemical analysis, particularly in micellar solutions, is well-known for its ability to solubilize organic molecules involved in redox processes that often simulate biological systems. Two primary effects influence the electrode reaction when surfactants are present: first, they can stabilize radical ions and intermediates, fundamentally modifying the reaction pathway; second, the adsorption of surfactant molecules alters the structure of the electrical double layer and the rate constant of charge transfer [28].

These alterations manifest as shifts in the step or peak potentials and generally enhance analytical characteristics, notably improving the reversibility of the electrochemical reaction and boosting detection sensitivity [29].

**Table 1:** Comparison of electrochemical parameters for REV at ZnO-NPs/CPE with different types of surfactants

Electrode system	Volume of surfactant added in µL	Current µA
ZnO–NPs/CPE	–	0.36
ZnO–NPs/CPE + SDS	100	0.40
	750	0.64
ZnO–NPs/CPE + CTAB	100	0.17
	500	0.13
	750	0.07

### Oxidation mechanism of REV

The electrochemical oxidation of REV at the ZnO-NP/CPE proceeds via a two-step mechanism.The linear Ep–pH relationship exhibits a slope of 35.8 mV per pH unit, consistent with a proton-coupled electron transfer process corresponding to an overall two-electron, one-proton reaction Fig. 5, in agreement with standard Nernstian behavior [30].

The oxidation is initiated by a one-electron transfer at the tertiary amine to form an amine radical cation, followed by α-deprotonation and a second one-electron oxidation to generate the corresponding iminium cation. Such stepwise radical-cation pathways are well established for tertiary amines [31], with the iminium species acting as a key electrophilic intermediate (Table 2).

**Fig. 8:**
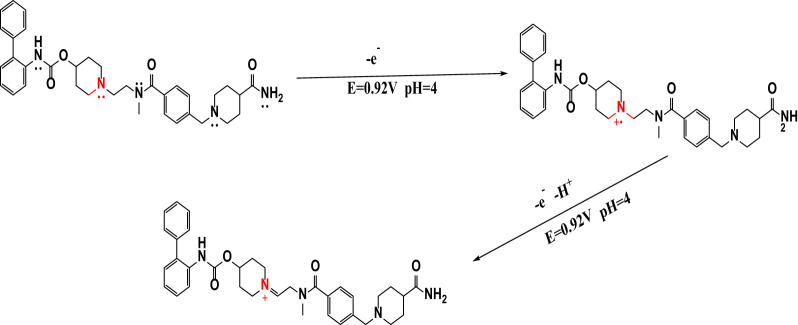
The suggested oxidation mechanism of REV on ZnO–NPs/CPE at B-R buffer pH 4

The anodic peak observed at 0.92 V in Britton–Robinson buffer at pH 4.0 is therefore attributed to the electrocatalytic oxidation of REV at the ZnO-NP-modified carbon paste electrode (Fig. 8).

**Table 2:** Mulliken partial charges of REV show the percentages of negative charge for the more electroactive site in REV structure, with hydrogen atoms added

Atom	Atom type	Mulliken charge	MMFF94 charge	Chemical environment	Susceptibility to oxidation
N (5)	N Amine	− 0.5358	− 0.81	Piperidine ring nitrogen attached to the alkyl chain and the methylamine bridge.	- Primary site for oxidative - High negative charge - Electron-rich

### Quantification of REV in its bulk material

**Fig. 9:**
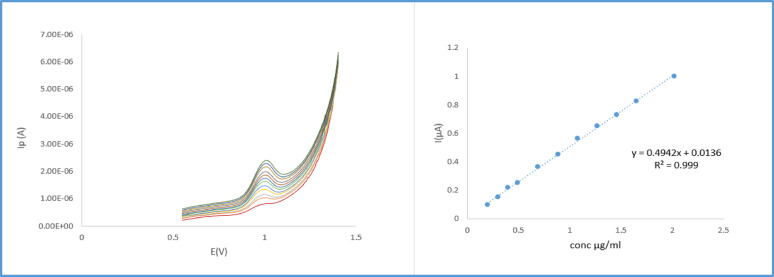
DPV of REV at ZnO–NPs/CPE at sweep rate = 0.06 Vs^−1^, pulse amplitude = 50 mV, pulse time = 0.04 s, voltage step = 0.06 V and voltage step time = 0.1 s in the presence of (SDS) and the corresponding calibration plots (n = 6)

 Voltammograms of differential pulse voltammetric determination of REV at ZnO–NPs/CPE in the presence of anionic surfactants SDS were obtained at 0.1 V/S scan rate with an applied potential range from 0.0 to 1.4 V. Significantly, the anodic signal manifested as one distinct peak appearing at approximately a voltage of 0.9–1.1 V measured by µA. To determine the quantity of REV in solution a linear plot is generated by correlating the anodic peak currents with the respective REV concentrations. with concentrations range of REV 0.19–2.0 µg mL^−1^ (Fig. 9). The statistical parameters demonstrated in Table 3.

**Table 3:** Statistical analysis for REV differential pulse voltammetry using ZnO–NPs/CPE with SDS at pH 4

Linearity range µg mL^−1^	(0.19–2.0)
Equation	I(µA) = 0.4942 conc µg mL^−1^ + 0.0136
Intercept	0.0136
Slope	0.4942
R^2^	0.999
Standard Error	0.00656
RSD	1.17
LOD^a^	0.04
LOQ^b^	0.13

*Obtained from an average of six experiments

^a^LOD = 3.3 σ/S

^b^LOQ = 10 σ/S

*σ* standard deviation of the regression residuals; *S* slope of linearity

###  Method validation [32]

#### Linearity and range

In the present work, a linear and direct correlation between the maximum current I in µA and the REV concentration in µg mL^−1^ was established through voltammetric analysis with ZnO–NPs/CPE. The linearity was the foundation for quantitative REV determination in the sample solution, enabling reproducible and sensitive detection within the adopted analytical method. The data are provided in Table 2. while graphical representation of calibration curve is shown in Fig. 9.

#### Minimum detectable concentration (LOD) and the lowest measurable concentration (LOQ)

The establishment of these parameters of the analytes were done according to (ICH) Q2(R1) guidelines [33] for analytical method validation. Specifically, LOD and LOQ were calculated from the standard deviation (σ) of response (generally derived from the blank or lowest concentration samples) and the slope (S) of the calibration curve solution prepared [34]. This statistical technique ensures that the values are indicative of the intrinsic precision and sensitivity of the analytical method established (Table 3).

#### Accuracy

Accuracy was expressed in terms of the calculated mean percent recovery. The calibration curves and the associated regression formulas were used to calculate the accuracy of four varied REV amounts, and the result demonstrated in Table 4.

**Table 4:** Accuracy results for the determination of REV by the proposed electrochemical method

Taken µg mL^−1^	Found* µg mL^−1^	Recovery %
0.35	0.347	99.14
0.52	0.518	99.62
0.92	0.921	100.11
1.51	1.53	101.32
Mean		100.05
SD		0.94
Variance		0.88

*Obtained from an average of six experiments

#### Repeatability (intra-day)

Six distinct measurements were taken within one day for each of the three chosen concentrations (0.25, 0.35, 1.5) µg mL^−1^ of REV were performed. Precision for the suggested method was quantified by calculating the percentage relative standard deviation %RSD, then the intermediate precision was verified by repeating the measurements over three sequential days. The Results from the precision assessment are presented in Table 5. The method demonstrated high precision, indicated by the percent relative standard deviation % RSD values for both repeatability and intermediate precision falling below 2%. optimal electrodes reproducibility was confirmed by creating 6 (ZnO-NPs/CPE) electrodes under equivalent conditions. These electrodes yielded nearly identical responses, reflected by a low %RSD value of 0.91.

**Table 5:** Precision analysis by % RSD for the differential pulse voltammetric determination of REV at ZnO–NPs/CPE

	% RSD
Repeatability of the peak current I(µA)	1.31
Intermediate precision of the peak current I(µA)	1.52
Reproducibility of the peak current I(µA)	0.91
Repeatability of the peak potential E(v)	1.20
Reproducibility of the peak potential E(v)	1.25

### Application to pharmaceutical dosage form

The proposed analytical methodology for the quantification of REV within its inhalation solution was evaluated utilizing a simulated formulation. This laboratory-prepared mimetic solution possessing active pharmaceutical ingredient, excipients and physicochemical properties (pH, ionic strength) of the commercial product, served as a representative matrix for method development and validation. The data collected using the suggested methods and the established method [5] were subjected to statistical comparison using student’s t-test and variance ratio f-test reveals no statistically significant difference between the two methods (Table 6).

**Table 6:** The suggested method is compared to the reported HPLC analysis for the determination of REV in simulated dosage form

Conc. taken µg mL^−1^	% Found*	
	The proposed DPV method	HPLC reported method [5]
0.25	100.20	100.17
0.35	100.10	99.97
1.5	99.55	100.30
Mean	99.95	100.15
Variance	0.12	0.03
F-Test	4.43 (19)	
t-test	0.21 (2.78)	

*Each result is the mean value for three separate determinations

### Application to spiked human urine

REV was successfully quantified in urine. Percentage of REV after a single oral dose of 200 µg was found to be 66.8% feces–0.32% urine [35]. Therefore, the existing methods are insufficient for measuring the ceiling concentration of the REV molecule excreted in urine as the amount of REV is outside the detection limits of the previously reported methods, as it evidenced by the data in Table 7. These findings establish the necessity for the proposed analytical approach. Interestingly, the developed procedure resulted in excellent and ideal recovery efficiencies of REV in urine as presented in Table 8. The analytical peak’s potential region remained clean across all tested matrices, showing no presence of interfering oxidation compounds or unwanted noise spikes.

**Table 7:** Comparison of the proposed method and other reported methods for determination of REV

Analytical method	Linearity	Application	Reference
RP-HPLC method	50–150 µg mL^−1^	Pharmaceutical Dosage form	[5]
Electrochemical Determination	0.19–2.0 µg mL^−1^	Pharmaceutical Dosage form&urine	suggested DPV voltammetric method

**Table 8:** Analytical performance data of spiked urine at ZnO–NPs/CPE

Added µg mL^−1^	Found *µg mL^−1^	Recovery %
0.25	0.248	99.20
0.35	0.355	101.43
0.95	0.958	100.84
1.8	1.79	99.44
Mean		100.23
SD		1.08
Variance		1.16

*Obtained from an average of six experiments

## Greenness assessment of the developed methods

A complete evaluation of environmental impact was performed for the developed voltammetric method by two assessment tools: GAPI [36] and AGREE [37]. The use of a combination of different tools provides precise and reliable results about greenness as each tool has its advantages and dis advantages and unique protocol [38]. The performance of developed and reported method was evaluated b using AGREE calculator. The developed voltametric method performed an AGREE score of (0.85) which shows excellent greenness than reported (0.73) HPLC method (Tables 9, 10, 11).

**Table 9:** Performance assessment of the developed voltammetric method and HPLC method by different greenness tools (GAPI, AGREE)

Tool	Developed voltametric method	Reported HPLC [5]
GAPI		
AGREE		

**Table 10:** Assessment of the greenness of the developed voltammetric method and HPLC method using AGREE calculator

Parameters	Developed voltametric method	Reported HPLC [5]
Select the sampling procedure	off-line	off-line
Enter the amount of sample in gm. or mL	0.015 g	0.05 g
What’s the positioning of the analytical device	Off-line	Off-line
How many major distinct steps are there in the sample preparation procedure? (sonication, mineralization, centrifugation, derivatization, etc.)	3 or fewer	3 or fewer
Degree of automation, sample preparation	Semi-automatic miniaturized	Semi-automatic, Not miniaturized
Select derivatization agent (if used)	Not used	Not used
Enter the amount of waste in g or mL	< 0.1 g	< 0.1 g
Number of analytes determined in a single run-sample throughput (sample analyzed per hour)	1 (analytes) 60	1(analytes) 4
Select the most energy-intensive technique used in the method Total power consumption of a single analysis in kWh	< 0.001 kWh	0.1–1.5 kWh
Select the type of reagent	No reagent	All reagents are bio-based
Does the method involve the use of toxic reagents of solvents	No	Yes Acetonitrile – perchloric acid
Select the threats which are not avoided	No threats	Highly flammable

**Table 11:** Assessment of the greenness of the developed voltammetric method and HPLC method using GAPI tool

Parameters	Developed voltametric method	Reported HPLC [5]
5-Type of method/analysis	Simple procedure	Simple procedure
(9) Amount	< 10 mL	< 10 mL
(10) Health hazard	NFPA score = 0	Moderate toxic NFPA score = 2
(11) Safety hazard	NFPA flammability is 0	Highest NFPA flammability or instability score of 2 or 3, or a special hazard is used.
(12) Energy	≤ 0.1 kWh per sample	≤ 1.5 kWh per sample
(13) Occupational hazard	Hermetic sealing of analytical process	Hermetic sealing of analytical process
(14) Waste	1–10 mL	> 10 mL
(15) Waste treatment - type of analsis	No treatment Qualitative and quantitative	No treatment Qualitative and quantitative

## Conclusions

We present a novel, fully validated voltammetric procedure for the fast and reliable determination of REV across diverse matrices, including pure substance, drug formulations and biofluids. This procedure employs a unique working electrode: a carbon paste modified with zinc oxide nanoparticles.

The inclusion of SDS in the electrode composition boosts electrical conductivity and enabling high performance. This new sensor system provides a crucial advancement over existing techniques by achieving demonstrably lower limits of detection and quantification compared to previous chromatographic methods (Table 6). This validated procedure represents a notably greener and more sustainable analytical choice (Table 9), requiring minimal sample preparation and drastically reducing the consumption of expensive, toxic organic solvents typically required by chromatographic methods.

The validation results were highly satisfactory, confirming the method’s utility for standard quality control within the pharmaceutical sector.

## Data Availability

Most data generated or analyzed during this study are included in this published.
